# Supplementary material for: Content-rich biological network constructed by mining PubMed abstracts
Source: BMC Bioinformatics. 2004 Oct 8;5:147. doi: 10.1186/1471-2105-5-147 (PMC528731; doi:10.1186/1471-2105-5-147)
Supplement: Additional File 2 — The original results of the above study (non-essential files are deleted to keep the file size under the limit set by BMC bioinformatics). [file 1471-2105-5-147-S2.bz2 › chilibotAdditionalFile2/dip10/341ID8076595E4256/html/TNFSF4_TNFRSF4.html]

 


 **TNFSF4** and **TNFRSF4** 
  
Found 114 abstracts in PubMed, retrieved 10.  
 

 What does Google say? 
 PDF only 
| .edu only 

---

**Interactive relationship** (e.g. stimulation, inhibition, etc)

**Neutral relationship**- In already sensitized animals, blocking OX40  [ **TNFRSF4** ]  OX40L  [ **TNFSF4** ]  interactions at the time of inhalation of aerosolized antigen suppressed memory effector accumulation in lung draining lymph nodes and lung, and prevented eosinophilia, airway hyperreactivity, mucus secretion, and Th2 cyto kine production.  Ref: 12860930 J Exp MedJ Exp Med,
- Thus, OX40  [ **TNFRSF4** ]  OX40L  [ **TNFSF4** ]  interactions are pivotal to the efficiency of recall responses regulated by memory Th2 cells.  Ref: 12860930 J Exp MedJ Exp Med,
- Interaction of OX40  [ **TNFRSF4** ]  CD134  [ **TNFRSF4** ]  and its ligand, OX40L  [ **TNFSF4** ] , has been implicated in T cell activation and cell migration.  Ref: 12794142 J Immunol, 2003
- Interaction of OX40  [ **TNFRSF4** ]  CD134  [ **TNFRSF4** ]  on T cells with its ligand OX40L  [ **TNFSF4** ]  on antigen presenting cells has been implicated in pathogenic T cell activation.  Ref: 12631559 Am J Physiol Gastrointest Liver Physiol, 2003
- These results suggest that the OX40  [ **TNFRSF4** ]  OX40L  [ **TNFSF4** ]  system is crucial for induction of the allogeneic T cell response and the OX40  [ **TNFRSF4** ]  OX40L  [ **TNFSF4** ]  system is subsequent to and dependent on CD28 signalling, but is crucial for the end outcome of the human alloreactive T cell response.  Ref: 12757617 Immunology, 2003

**Non-interactive relationship** (e.g. studied together, co-existance, homology, etc.)

- This study was performed to explore the involvement of OX40  [ **TNFRSF4** ]  OX40L  [ **TNFSF4** ]  in the development of T cell mediated chronic colitis.  Ref: 12631559 Am J Physiol Gastrointest Liver Physiol, 2003
- In this study, we show that engagement of OX40  [ **TNFRSF4** ]  by an OX40L  [ **TNFSF4** ]  Ig fusion protein drives IFN gamma production by CD4 T cells and reduces eosinophilia and C. neoformans burden in the lung.  Ref: 12794142 J Immunol, 2003
- Our present results suggested a pivotal role of OX40  [ **TNFRSF4** ]  OX40L  [ **TNFSF4** ]  in the pathogenesis of T cell mediated chronic colitis.  Ref: 12631559 Am J Physiol Gastrointest Liver Physiol, 2003
- However, B cell costimulation via the OX40L  [ **TNFSF4** ]  OX40  [ **TNFRSF4** ]  pathway that has been implicated in CD4 cell expansion, survival, and Th2 development was required.  Ref: 12668647 J Exp Med, 2003
- We first demonstrated that anti OX40  [ **TNFRSF4** ]  ligand  [ **TNFSF4** ]  anti OX40L  [ **TNFSF4** ]  monoclonal antibody mAb could markedly suppress the mixed leucocyte reaction MLR of peripheral blood mononuclear cells PBMC.  Ref: 12757617 Immunology, 2003
- We recently demonstrated a critical contribution of OX40  [ **TNFRSF4** ]  ligand  [ **TNFSF4** ]  OX40L  [ **TNFSF4** ]  to the development of Th2 mediated experimental leishmaniasis.  Ref: 12672051 Eur J Immunol, 2003
- Unlike dendritic cells, the CD4 CD3 cells have little CD80 or CD86 expression but do express high levels of the TNF ligands, OX40  [ **TNFRSF4** ]  ligand  [ **TNFSF4** ]  and CD30 ligand.  Ref: 12753741 Immunity, 2003
- We investigated the effect of CD134  [ **TNFRSF4** ]  CD134L  [ **TNFSF4** ]  pathway blockade on allograft rejection in fully MHC mismatched rat cardiac and skin transplantation models.  Ref: 12626546 J Immunol, 2003
- The CD134  [ **TNFRSF4** ]  CD134  [ **TNFRSF4** ]  ligand  [ **TNFSF4** ]  CD134L  [ **TNFSF4** ]  costimulatory pathway has been shown to be critical for both T and B cell activation.  Ref: 12626546 J Immunol, 2003
- To further define the contribution of the OX40  [ **TNFRSF4** ]  OX40L  [ **TNFSF4** ]  system to the MLR, we set up a co culture system of CD4 T cells and allogeneic monocyte derived dendritic cells DCs.  Ref: 12757617 Immunology, 2003
- Critical role for OX40  [ **TNFRSF4** ]  ligand  [ **TNFSF4** ]  in the development of pathogenic Th2 cells in a murine model of asthma.  Ref: 12672051 Eur J Immunol, 2003
- Our data suggest that CD134  [ **TNFRSF4** ]  CD134L  [ **TNFSF4** ]  is a critical pathway in alloimmune responses, especially recall primed responses, and is synergistic with CD28 B7 in mediating T cell effector responses during allograft rejection.  Ref: 12626546 J Immunol, 2003
- The Role of the CD134  [ **TNFRSF4** ]  CD134  [ **TNFRSF4** ]  Ligand  [ **TNFSF4** ]  Costimulatory Pathway in Alloimmune Responses In Vivo.  Ref: 12626546 J Immunol, 2003
